# Heterogeneous Differential Evolution for Numerical Optimization

**DOI:** 10.1155/2014/318063

**Published:** 2014-02-05

**Authors:** Hui Wang, Wenjun Wang, Zhihua Cui, Hui Sun, Shahryar Rahnamayan

**Affiliations:** ^1^School of Information Engineering, Nanchang Institute of Technology, Nanchang 330099, China; ^2^School of Business Administration, Nanchang Institute of Technology, Nanchang 330099, China; ^3^Complex System and Computational Intelligent Laboratory, Taiyuan University of Science and Technology, Taiyuan 030024, China; ^4^State Key Laboratory for Novel Software Technology, Nanjing University, Nanjing 210023, China; ^5^Department of Electrical, Computer and Software Engineering, University of Ontario Institute of Technology, 2000 Simcoe Street North Oshawa, ON, Canada L1H 7K4

## Abstract

Differential evolution (DE) is a population-based stochastic search algorithm which has shown a good performance in solving many benchmarks and real-world optimization problems. Individuals in the standard DE, and most of its modifications, exhibit the same search characteristics because of the use of the same DE scheme. This paper proposes a simple and effective heterogeneous DE (HDE) to balance exploration and exploitation. In HDE, individuals are allowed to follow different search behaviors randomly selected from a DE scheme pool. Experiments are conducted on a comprehensive set of benchmark functions, including classical problems and shifted large-scale problems. The results show that heterogeneous DE achieves promising performance on a majority of the test problems.

## 1. Introduction

Differential evolution (DE) [1] is a well-known algorithm for global optimization over continuous search spaces. Although DE has shown a good performance over many optimization problems, its performance is greatly influenced by its mutation scheme and control parameters (population size, scale factor, and crossover rate). To enhance the performance of DE, many improved DE variants have been proposed based on modified mutation strategies [2, 3] or adaptive parameter control [4–6].

The standard DE and most of its modifications [7–10] make use of homogeneous populations where all of the individuals follow exactly the same behavior. That is, individuals implement the same DE scheme, such as DE/rand/1/bin and DE/best/1/bin. The effect is that individuals in the population behave with the same exploration and/or exploitation characteristics [11]. An ideal optimization algorithm should balance exploration and exploitation during the search process. Initially, the algorithm should concentrate on exploration. As the iteration increases, it would be better to use exploitation to find more accurate solutions. However, it is difficult to determine when the algorithm should switch from an explorative behavior to an exploitative behavior. To tackle this problem, a new concept of heterogeneous swarms [11] is proposed and applied to particle swarm optimization (PSO) [12, 13], where particles in the swarm use different velocity and position update rules. Therefore, the swarm may consist of explorative particles as well as exploitative particles. This makes the heterogeneous PSO have the ability to balance exploration and exploitation during the search process.

In this paper, a simple and effective heterogeneous DE (HDE) algorithm is proposed inspired by the idea of heterogeneous swarms [11]. In HDE, individuals will be allocated to different search behaviors randomly selected from a DE scheme pool. However, the concept of heterogeneous swarms used in DE is not new. Qin et al. [6] proposed a self-adaptive DE (SaDE), where individuals are also allowed to implement different mutation schemes according to a complex probability model. Gong et al. [14] combined a strategy adaptation mechanism with four mutation strategies proposed in JADE [3]. For other self-adaptive DE variants [8], the search characteristics of individuals dynamically change according to the adaptation of the control parameters (scale factor and/or crossover rate). The HDE proposed in this paper differs from the above DE variants. In HDE, the behaviors are randomly assigned from a DE scheme pool. By the suggestions of heterogeneous PSO [11], two heterogeneous models are proposed. This first one is static HDE (sHDE), where the randomly selected behaviors are fixed during the evolution. The second one is dynamic HDE (dHDE), where the behaviors can randomly change during the search process. Experimental studies are conducted on a comprehensive set of benchmark functions, including classical problems and shifted large-scale problems. Simulation results demonstrate the efficiency and effectiveness of the proposed heterogeneous DE.

The rest of the paper is organized as follows. In Section 2, the standard DE algorithm is briefly introduced. The HDE is proposed in Section 3. Experimental simulations, results, and discussions are presented in Section 4. Finally, the work is concluded in Section 5.

## 2. Differential Evolution

There are several variants of DE [1], which use different mutation strategies and/or crossover schemes. To distinguish these different DE schemes, the notation “DE/*x*/*y*/*z*” is used, where “DE” indicates the DE algorithm, “*x*” denotes the vector to be mutated, “*y*” is the number of difference vectors used in the mutation, and “*z*” stands for the type of crossover scheme, exponential (exp) or binomial (bin). The following section discusses the mutation and crossover operations.

### 2.1. Mutation

For each vector *X*_*i*,*G*_ at generation *G*, this operation creates mutant vectors *V*_*i*,*G*_ based on the current parent population. The following are five well-known variant mutation strategies:

(1) DE/rand/1
  (1) $$\begin{array}{c} V_{i,G}=X_{i_{1},G}+F\cdot \left(X_{i_{2},G}-X_{i_{3},G}\right), \end{array}$$
(2) DE/best/1
  (2) $$\begin{array}{c} V_{i,G}=X_{\text{best},G}+F\cdot \left(X_{i_{1},G}-X_{i_{2},G}\right), \end{array}$$
(3) DE/current-to-best/1
  (3) $$\begin{array}{c} V_{i,G}=X_{i,G}+F\cdot \left(X_{\text{best},G}-X_{i,G}\right)+F\cdot \left(X_{i_{1},G}-X_{i_{2},G}\right), \end{array}$$
(4) DE/rand/2
  (4) $$\begin{array}{c} V_{i,G}=X_{i_{1},G}+F\cdot \left(X_{i_{2},G}-X_{i_{3},G}\right)+F\cdot \left(X_{i_{4},G}-X_{i_{5},G}\right), \end{array}$$
(5) DE/best/2
  (5) $$\begin{array}{c} V_{i,G}=X_{\text{best},G}+F\cdot \left(X_{i_{1},G}-X_{i_{2},G}\right)+F\cdot \left(X_{i_{3},G}-X_{i_{4},G}\right), \end{array}$$

where the indices *i*_1_, *i*_2_, *i*_3_, *i*_4_, and *i*_5_ are mutually different random indices chosen from the set {1,2,…, *N*_*p*_}, *N*_*p*_ is the population size, and all are different from the base index *i*. The scale factor *F* is a real number that controls the difference vectors. *X*_best,*G*_ is the best vector in terms of fitness value at the current generation *G*.

### 2.2. Crossover

Similar to EAs, DE also employs a crossover operator to build trial vectors *U*_*i*,*G*_ by recombining the current vector *X*_*i*,*G*_ and the mutant one *V*_*i*,*G*_. The trail vector is defined as follows:

(6) $$\begin{array}{c} u_{i,j,G}=\{\begin{array}{cc} v_{i,j,G}, & \text{if}\;{\text{rand}}_{j}\left(0,1\right)\leq \text{CR}\vee j=j_{\text{rand}}\\ x_{i,j,G}, & \text{otherwise}, \end{array} \end{array}$$

where CR ∈ (0,1) is the predefined crossover probability, rand_*j*_(0,1) is a uniform random number within [0,1] for the *j*th dimension, and *j*_rand_ ∈ {1,2,…, *D*} is a random index.

### 2.3. Selection

After the crossover, a greedy selection mechanism is used to select the better one from the parent vector *X*_*i*,*G*_ and the trail vector *U*_*i*,*G*_ according to their fitness values *f*(·). Without losing of generality, this paper only considers minimization problems. If, and only if, the trial vector *U*_*i*,*G*_ is better than the parent vector *X*_*i*,*G*_, then *X*_*i*,*G*+1_ is set to *U*_*i*,*G*_; otherwise, we keep *X*_*i*,*G*+1_ the same with *X*_*i*,*G*_:

(7) $$\begin{array}{c} X_{i,G+1}=\{\begin{array}{cc} U_{i,G}, & \text{if}\;\;f\left(U_{i,G}\right)\leq f\left(X_{i,G}\right)\\ X_{i,G}, & \text{otherwise}. \end{array} \end{array}$$

## 3. Heterogeneous DE

In the standard DE and most of its modifications, individuals in the population behave with the same search characteristics, exploration, and/or exploitation, because of the use of the same DE scheme. The effect is that the algorithms could hardly balance exploration and exploitation during the search process. Inspired by the heterogeneous swarms [11], a simple and effective heterogeneous DE (HDE) algorithm is proposed in this paper. Compared to DE and most of its variants, individuals in HDE are allowed to implement different behaviors.

To implement different DE schemes in HDE, we need to address two questions. First, which DE scheme should be chosen to construct the DE scheme pool? Second, how do we assign DE schemes to individuals?

As mentioned before, there are several DE schemes, and different DE schemes have different search characteristics. In this paper, three different DE schemes are chosen to construct the DE scheme pool: (1) DE/rand/1/bin; (2) DE/best/1/bin; and (3) DE/BoR/1/bin [16]. The first two schemes are two basic DE strategies proposed in [1], and the last one was recently proposed in [16], where a new mutation strategy called “best-of-random” (BoR) is defined as follows:

(8) $$\begin{array}{c} V_{i,G}=X_{i_{b},G}+F\cdot \left(X_{i_{1},G}-X_{i_{2},G}\right), \end{array}$$

where the individuals *X*_*i*_1__, *X*_*i*_2__, and *X*_*i*_*b*__ are randomly chosen from the current population, *i* ≠ *i*_1_ ≠ *i*_2_ ≠ *i*_*b*_, and *X*_*i*_*b*__ is the best one of them; that is, *f*(*X*_*i*_*b*__) ≤ min⁡(*f*(*X*_*i*_1__), *f*(*X*_*i*_2__)).

There are two reasons for choosing these DE schemes. First, these three DE schemes are very simple and easy to implement. Second, each of them has different search characteristics. For the DE/rand/1/bin, it obtains higher population diversity. DE/best/1/bin shows faster convergence speed. The last one provides a middle phase between the first two DE schemes. Note that this paper only chooses three DE schemes to construct the DE scheme pool and other DE schemes can also be possibly used.

**Figure 1:**
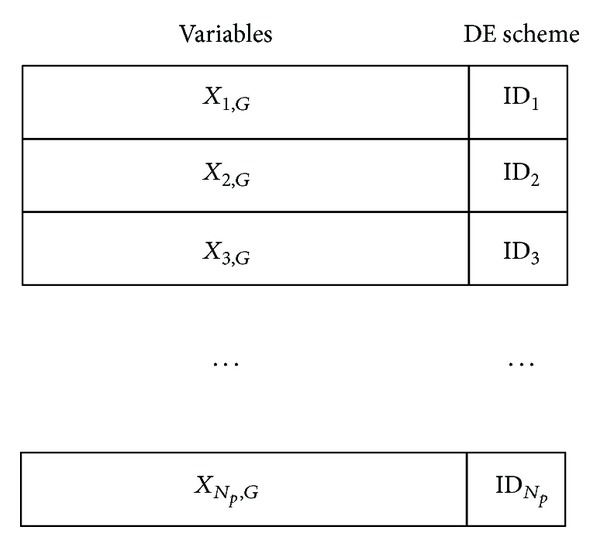
The encoding of individuals in heterogeneous DE.

Figure 1 presents the encoding method in HDE, where ID_*i*_ denotes the employed DE scheme for the *i*th individual. In this paper, ID_*i*_ ∈ {1,2, 3}; that is, ID_*i*_ = 1 indicates the DE/rand/1/bin scheme, ID_*i*_ = 2 denotes the DE/best/1/bin scheme, and ID_*i*_ = 3 stands for the DE/BoR/1/bin scheme.

In order to address the second question, two different heterogeneous models, namely, static HDE (sHDE) and dynamic HDE (dHDE), are used to assign DE schemes to individuals [11].

- In the static HDE (sHDE), DE schemes are randomly assigned to individuals in population initialization. The assigned DE schemes do not change during the search process.
- In the dynamic HDE (dHDE), DE schemes are randomly assigned to individuals during initialization. As the iteration increases, the assigned DE schemes of individuals are not fixed. They can randomly change during the search process. An individual randomly selects a new DE scheme from the DE scheme pool when the individual fails to improve its objective fitness value. In the standard DE and its variants, the objective fitness value of each individual *X*_*i*_ satisfies *f*(*X*_*i*,1_) ≤ *f*(*X*_*i*,2_) ≤ ⋯≤*f*(X_*i*,*G*_). If the new generated individual (trail vector *U*_*i*_) could not improve its previous position (*X*_*i*_), it may indicate early stagnation. This can be addressed by assigning a new DE scheme to the individual.

The main steps of dynamic heterogeneous DE (dHDE) are presented in Algorithm 1, where *P* is the current population, FEs is the number of fitness evaluations, and MAX_FEs is the maximum number of FEs. For static HDE (sHDE), please delete line 21 in Algorithm 1.

## 4. Experimental Verifications

This section provides experimental studies of HDE on 18 well-known benchmark optimization problems. According to the properties of these problems, two series of experiments are conducted: (1) comparison of HDE on classical optimization problems and (2) comparison of HDE on shifted large-scale optimization problems.

### 4.1. Results on Classical Optimization Problems

In this section, 12 classical benchmark problems are used to verify the performance of HDE. These problems were considered in [2, 5, 17–19].  Table 1 presents a brief description of these benchmark problems. All the problems are to be minimized.

Experiments are conducted to compare HDE with other six DE variants. The involved algorithms are listed below:

- DE/rand/1/bin,
- DE/best/1/bin,
- DE/BoR/1/bin [16],
- self-adapting DE (jDE) [5],
- DE with neighborhood search (NSDE) [20],
- DE using neighborhood-based mutation (DEGL) [2],
- the proposed sHDE and dHDE.

In the experiments, we have two series of comparisons: (1) comparison of sHDE/dHDE with basic DE schemes and (2) comparison of sHDE/dHDE with state-of-the-art DE variants. The first comparison aims to check whether the heterogeneous method is helpful to improve the performance of DE. The second comparison investigates whether the proposed approach is better or worse than some recently proposed DE variants.

For these two comparisons, we use the same parameter settings as follows. For the three basic DE schemes (DE/rand/1/bin, DE/best/1/bin, and DE/BoR/1/bin) and sHDE/dHDE, the control parameters, *F* and CR, are set to 0.5 and 0.9, respectively [5]. For jDE, NSDE, and DEGL, the parameters *F* and CR are self-adaptive. For all algorithms, the population size (*N*_*p*_) and maximum number of FEs are set to 10 · *D* and 5.00*E* + 05, respectively [2]. All the experiments are conducted 30 times, and the mean error fitness values are reported. 

#### 4.1.1. Comparison of HDE with Basic DE Schemes

The comparison results of HDE with the three basic DE schemes are presented in Table 2, where “Mean” denotes the mean error fitness values. From the results, sHDE and dHDE outperform other three basic DE schemes on a majority of test problems. For unimodal problems (*f*_1_ − *f*_4_), DE/best/1/bin shows faster convergence than other algorithms for the attraction of the global best individual. For *f*_6_ and *f*_7_, all the algorithms obtain similar performance. DE/rand/1/bin provides high population diversity but slows down convergence rate. That is why DE/rand/1/bin performs better than DE/best/1/bin on most multimodal problems, but worse on unimodal problems. DE/BoR/bin/1 is a middle phase of DE/rand/1/bin and DE/best/1/bin. It obtains better performance than the other two basic DE schemes. For the heterogeneity of these basic DE schemes, sHDE and dHDE significantly improve the performance of DE. For unimodal problems, DE/best/1/bin obtains the best performance, while sHDE and dHDE perform better than DE/rand/1/bin and DE/BoR/1/bin. For multimodal problems, DE/best/1/bin is the worst algorithm, while sHDE and dHDE obtain better performance than other algorithms.

For the comparison of sHDE and dHDE, sHDE performs better on 3 problems, while dHDE achieves better results on 5 problems. For the remaining 4 problems, both of them could search the global optimum. Although the dynamic heterogeneous model improves the performance of HDE on many problems, it may lead to premature convergence. In the dHDE, if the current individual could not improve its fitness value, a new DE scheme is assigned to the individual. If the new scheme is DE/best/1/bin, the individual will be attracted by the global best individuals. This will potentially run the risk of premature convergence.

#### 4.1.2. Comparison of HDE with Other State-of-the-Art DE Variants

The comparison results of HDE with other three state-of-the-art DE variants are presented in Table 3, where “Mean” denotes the mean error fitness values. From the results, sHDE and dHDE perform better than other three DE algorithms on the majority of test problems. dHDE outperforms jDE and NSDE on all test problems except for *f*_8_. On this problem, dHDE falls into the local minima, while sHDE could successfully solve it. dHDE achieves better results than DEGL on 9 problems, while DEGL performs better than dHDE on the remaining 3 problems.

In order to compare the performance of multiple algorithms on the test suite, we conducted Friedman test according to the suggestions of [21].  Table 4 shows the average ranking of jDE, NSDE, DEGL, sHDE, and dHDE. As seen, the performance of the five algorithms can be sorted by average ranking into the following order: dHDE, sHDE, DEGL, jDE, and NSDE. The best average ranking was obtained by the dHDE algorithm, which outperforms the other four algorithms.

### 4.2. Results of HDE on Shifted Large-Scale Optimization Problems

To verify the performance of HDE on complex optimization problems, this section provides experimental studies of HDE on 6 shifted large-scale problems. These problems were considered in CEC 2008 special session and competition on large-scale global optimization [15].  Table 5 presents a brief descriptions of these benchmark problems. All the problems are to be minimized.

Experiments are conducted to compare four DE algorithms including the proposed sHDE and dHDE on 6 test problems with *D* = 500. The involved algorithms are listed below:

- opposition-based DE (ODE) [22],
- modified DE using Cauchy mutation (MDE) [23],
- the proposed sHDE and dHDE.

To have a fair comparison, all the four algorithms use the same parameter settings by the suggestions of [22]. The population size *N*_*p*_ is set to *D*. The control parameters, *F* and CR, are set to 0.5 and 0.9, respectively [22]. For ODE, the rate of opposition *J*_*r*_ is 0.3. The maximum number of FEs is 5000 · *D*. All the experiments are conducted 30 times, and the mean error fitness values are reported.

Table 6 presents the comparison results of HDE with ODE and MDE on shifted large-scale problems. As seen, sHDE and dHDE outperform ODE and MDE on four problems. The static heterogeneous model slightly improves the final solutions of DE, while the dynamic model significantly improves the results on *F*_1_ and *F*_3_. However, the two heterogeneous models do not always work. For some problems, sHDE and/or dHDE perform worse than ODE and MDE. The possible reason is that the heterogeneous models may hinder the evolution. For the static model, the entire population is divided into three groups. Each group uses a different basic DE scheme to generate new individuals. Though these groups share the search information of the entire population, each group with small population may obtain slow convergence rate. As mentioned before, the dynamic heterogeneous model runs the risk of premature convergence.

## 5. Conclusion

In the standard DE and most of its modifications, individuals follow the same search behavior for using the same DE scheme. The effect is that the algorithms could hardly balance explorative and exploitative abilities during the evolution. Inspired by the heterogeneous swarms, a simple and effective heterogeneous DE (HDE) is proposed in this paper, where individuals could follow different search characteristics randomly selected from a DE scheme pool. To implement the HDE, two heterogeneous models are proposed, one static, where individuals do not change their search behaviors, and a dynamic model where individuals may change their search behaviors. Both versions of HDE initialize individual behaviors by randomly selecting DE schemes from a DE scheme pool. In the dynamic HDE, when an individual does not improve its fitness value, a new different DE scheme is randomly selected from the DE scheme pool.

To verify the performance of HDE, two types of benchmark problems, including classical problems and shifted large-scale problems, are used in the experiments. Simulation results show that the proposed sHDE and dHDE outperform the other eight DE variants on a majority of test problems. It demonstrates that the heterogeneous models are helpful to improve the performance of DE.

For the dynamic heterogeneous model, the frequency of changing new DE schemes may affect the performance of dHDE. More experiments will be investigated. The concept of heterogeneous swarms can be applied to other evolutionary algorithms to obtain good performance. Future research will investigate different algorithms based on heterogeneous swarms. Moreover, we will try to apply our approach to some real-world problems [24, 25].

## Figures and Tables

**Algorithm 1:**
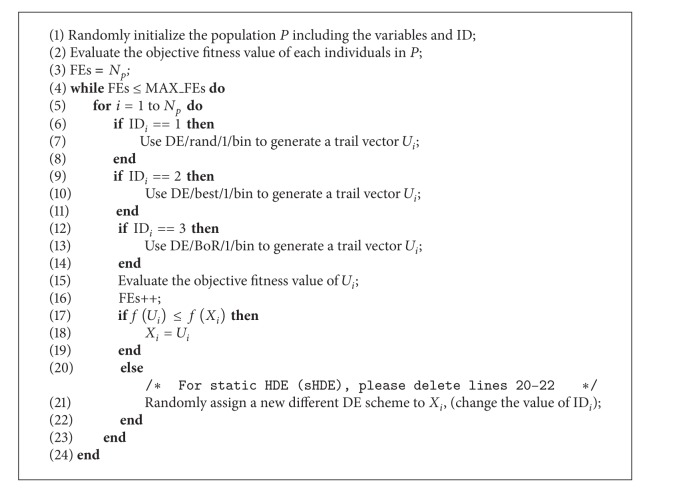
The dynamic heterogeneous DE (dHDE).

**Table 1:** The 12 classical benchmark optimization problems

Problem	Name	*D*	Properties	Search range
*f*_1_	Sphere	25	Unimodal	[−100,100]
*f*_2_	Schewefel 2.22	25	Unimodal	[−10,10]
*f*_3_	Schewefel 1.2	25	Unimodal	[−100,100]
*f*_4_	Schewefel 2.21	25	Unimodal	[−100,100]
*f*_5_	Rosenbrock	25	Multimodal	[−30,30]
*f*_6_	Step	25	Unimodal	[−100,100]
*f*_7_	Quartic with noise	25	Unimodal	[−1.28,1.28]
*f*_8_	Schewefel 2.26	25	Multimodal	[−500,500]
*f*_9_	Rastrigin	25	Multimodal	[−5.12,5.12]
*f*_10_	Ackley	25	Multimodal	[−32,32]
*f*_11_	Griewank	25	Multimodal	[−600,600]
*f*_12_	Penalized	25	Multimodal	[−50,50]

**Table 2:** Comparison of HDE with basic DE schemes on classical benchmark problems.

Problem	DE/rand/1/bin	DE/best/1/bin	DE/BoR/1/bin	sHDE	dHDE
	Mean	Mean	Mean	Mean	Mean
*f*_1_	0.00*E* + 00	4.38*E* − 27	2.37*E* − 46	6.58*E* − 79	0.00*E* + 00
*f*_2_	0.00*E* + 00	2.64*E* − 13	3.02*E* − 22	3.32*E* − 39	4.96*E* − 103
*f*_3_	2.68*E* − 125	2.41*E* − 04	1.51*E* − 11	3.10*E* − 21	7.21*E* − 57
*f*_4_	5.34*E* − 64	1.77*E* − 07	1.89*E* − 11	1.42*E* − 07	2.49*E* − 40
*f*_5_	5.75*E* − 29	2.91*E* − 04	1.45*E* − 16	1.11*E* − 29	1.73*E* − 29
*f*_6_	0.00*E* + 00	0.00*E* + 00	0.00*E* + 00	0.00*E* + 00	0.00*E* + 00
*f*_7_	1.38*E* − 03	2.50*E* − 03	1.52*E* − 03	8.31*E* − 04	1.84*E* − 03
*f*_8_	3.36*E* + 03	1.68*E* + 03	2.78*E* + 03	0.00*E* + 00	8.56*E* + 02
*f*_9_	4.38*E* + 01	1.32*E* + 02	2.62*E* + 01	5.22*E* + 01	0.00*E* + 00
*f*_10_	2.81*E* + 00	2.55*E* − 14	4.14*E* − 15	4.14*E* − 15	4.14*E* − 15
*f*_11_	2.95*E* − 02	0.00*E* + 00	0.00*E* + 00	0.00*E* + 00	0.00*E* + 00
*f*_12_	9.96*E* − 01	5.00*E* − 28	1.88*E* − 32	1.88*E* − 32	1.88*E* − 32

**Table 3:** Comparison of HDE with jDE, NSDE, and DEGL on classical benchmark problems.

Problem	jDE	NSDE	DEGL	sHDE	dHDE
	Mean	Mean	Mean	Mean	Mean
*f*_1_	4.04*E* − 35	9.55*E* − 35	8.78*E* − 37	6.58*E* − 79	0.00*E* + 00
*f*_2_	8.34*E* − 26	8.94*E* − 30	4.95*E* − 36	3.32*E* − 39	4.96*E* − 103
*f*_3_	4.76*E* − 14	3.06*E* − 09	1.21*E* − 26	3.10*E* − 21	7.21*E* − 57
*f*_4_	3.02*E* − 14	2.09*E* − 11	4.99*E* − 15	1.42*E* − 04	2.49*E* − 40
*f*_5_	5.64*E* − 26	2.65*E* − 25	6.89*E* − 25	1.11*E* − 29	1.73*E* − 29
*f*_6_	1.67*E* − 36	4.04*E* − 28	9.56*E* − 48	0.00*E* + 00	0.00*E* + 00
*f*_7_	3.76*E* − 02	4.35*E* − 03	1.05*E* − 07	8.31*E* − 04	1.84*E* − 03
*f*_8_	0.00*E* + 00	2.60*E* + 00	0.00*E* + 00	0.00*E* + 00	8.56*E* + 02
*f*_9_	6.74*E* − 24	4.84*E* − 21	5.85*E* − 25	5.22*E* + 01	0.00*E* + 00
*f*_10_	7.83*E* − 15	5.97*E* − 10	5.98*E* − 23	4.14*E* − 15	4.14*E* − 15
*f*_11_	1.83*E* − 28	7.93*E* − 26	2.99*E* − 36	0.00*E* + 00	0.00*E* + 00
*f*_12_	9.37*E* − 24	5.85*E* − 21	7.21*E* − 27	1.88*E* − 32	1.88*E* − 32

**Table 4:** Average rankings achieved by Friedman test.

Algorithms	Ranking
dHDE	4.17
sHDE	3.58
DEGL	3.50
jDE	2.25
NSDE	1.50

**Table 5:** The 6 shifted large-scale benchmark optimization problems proposed in [15].

Problem	Name	*D*	Properties	Search range
*F*_1_	Shifted Sphere	500	Unimodal, separable, scalable	[−100,100]
*F*_2_	Shifted Schewefel 2.21	500	Unimodal, nonseparable	[−100,100]
*F*_3_	Shifted Rosenbrock	500	Multimodal, nonseparable	[−100,100]
*F*_4_	Shifted Rastrigin	500	Multimodal, separable	[−5,5]
*F*_5_	Shifted Griewank	500	Multimodal, nonseparable	[−600,600]
*F*_6_	Shifted Ackley	500	Multimodal, separable	[−32,32]

**Table 6:** Comparison of HDE with ODE and MDE on shifted large-scale benchmark problems.

Problem	ODE	MDE	sHDE	dHDE
	Mean	Mean	Mean	Mean
*F*_1_	8.02*E* + 01	1.95*E* + 01	8.45*E* + 00	1.14*E* − 10
*F*_2_	5.78*E* + 00	2.70*E* + 01	8.54*E* + 01	1.01*E* + 02
*F*_3_	1.54*E* + 05	4.67*E* + 05	1.52*E* + 05	1.29*E* + 03
*F*_4_	4.22*E* + 03	4.14*E* + 03	5.27*E* + 03	3.42*E* + 03
*F*_5_	1.77*E* + 00	1.52*E* + 00	6.94*E* − 01	1.83*E* − 01
*F*_6_	4.51*E* + 00	4.02*E* + 00	1.26*E* + 00	1.58*E* + 01
